# Biogeography and evolution of *Thermococcus* isolates from hydrothermal vent systems of the Pacific

**DOI:** 10.3389/fmicb.2015.00968

**Published:** 2015-09-24

**Authors:** Mark T. Price, Heather Fullerton, Craig L. Moyer

**Affiliations:** Department of Biology, Western Washington UniversityBellingham, WA, USA

**Keywords:** *Thermococcus*, hydrothermal vents, subsurface microbiology, biogeography, evolution

## Abstract

*Thermococcus* is a genus of hyperthermophilic archaea that is ubiquitous in marine hydrothermal environments growing in anaerobic subsurface habitats but able to survive in cold oxygenated seawater. DNA analyses of *Thermococcus* isolates were applied to determine the relationship between geographic distribution and relatedness focusing primarily on isolates from the Juan de Fuca Ridge and South East Pacific Rise. Amplified fragment length polymorphism (AFLP) analysis and multilocus sequence typing (MLST) were used to resolve genomic differences in 90 isolates of *Thermococcus*, making biogeographic patterns and evolutionary relationships apparent. Isolates were differentiated into regionally endemic populations however there was also evidence in some lineages of cosmopolitan distribution. The biodiversity identified in *Thermococcus* isolates and presence of distinct lineages within the same vent site suggests the utilization of varying ecological niches in this genus. In addition to resolving biogeographic patterns in *Thermococcus*, this study has raised new questions about the closely related *Pyrococcus* genus. The phylogenetic placement of *Pyrococcus* type strains shows the close relationship between *Thermococcus* and *Pyrococcus* and the unresolved divergence of these two genera.

## Introduction

Microorganisms constitute the majority of known life forms making up greater than two-thirds of the metabolic and genetic diversity of the planet (Whitaker, 2006). The evolutionary forces responsible for shaping this vast amount of microbial diversity have been a topic of debate. Historically, microorganisms were viewed as having panmictic distributions, being constrained only by environmental conditions (Baas-Becking, 1934). Contemporary research has shown that limited dispersal, geographic isolation, selection and genetic drift can lead to divergence in microbial populations (Cho and Tiedje, 2000). The significance of physical isolation on the population divergence of terrestrial hyperthermophiles has been well documented (Papke et al., 2003; Whitaker et al., 2003; Papke and Ward, 2004; Whitaker, 2006; Reno et al., 2009); however, it is unknown whether similar patterns of isolation can be observed in hyperthermophiles at marine hydrothermal vents or if there is greater dispersal in these marine environments.

Marine hydrothermal vents are unique environments with extreme chemical, nutrient and temperature gradients. The organisms inhabiting hydrothermal vents are dispersed along plate boundaries and can be separated from other vent systems by thousands of kilometers creating island-like ecosystems. While there has been evidence in support of endemic community structure at hydrothermal vents (Opatkiewicz et al., 2009; Flores et al., 2012), descriptions of biogeography for individual microbial taxa have been uncommon (Escobar-Páramo et al., 2005; DeChaine et al., 2006; McAllister et al., 2011; Mino et al., 2013). The limited descriptions of microbial biogeography at hydrothermal vents can be mitigated through the isolation of microorganisms in culture and sequencing of individual genomes (Ramette and Tiedje, 2007; Meyer and Huber, 2014).

The hyperthermophilic archaea of the *Thermococcales* order are found at hydrothermal vents and can serve as model organisms for the study of biogeography and evolution. *Thermococcales* belong to the euryarchaeota phylum and consist of the three genera *Pyrococcus* (Fiala and Stetter, 1986), *Thermococcus* (Zillig et al., 1983), and *Paleococcus* (Takai et al., 2000). The genera *Thermococcus* and *Pyrococcus* are commonly found at hydrothermal vent systems and are readily isolated in culture allowing for comparisons among individual isolates (Jannasch et al., 1992; Erauso et al., 1993; Atomi et al., 2004; Bae et al., 2006; Teske et al., 2009). Genomic and phenotypic differences between *Thermococcus* and *Pyrococcus* delineate these two closely related genera, e.g., *Pyrococcus* has a higher optimum growth temperature (Garrity et al., 2001). Known *Thermococcus* isolates have a maximum growth temperature of 90°C, but some species have been shown to grow at temperatures as low as 45°C (Adams, 1993; Robb and Place, 1995; Summit and Baross, 1998). However, between these two genera, *Thermococcus* has the highest number of characterized isolates (Garrity et al., 2001; Holden et al., 2001; Teske et al., 2009; Zivanovic et al., 2009).

*Thermococcus* exhibit a variety of metabolic strategies; however, the majority are anaerobic heterotrophs that ferment organic compounds (Robb and Place, 1995; Teske et al., 2009). The addition of elemental sulfur enhances growth in *Thermococcus* and is required in some strains (Teske et al., 2009). Lithotrophic metabolic pathways have been shown as well, e.g., *T. onnurines* and other type strains, can oxidize carbon monoxide to CO_2_ using carbon monoxide dehydrogenases or CODHs (Lee et al., 2008; Sokolova et al., 2009; Zivanovic et al., 2009; Oger et al., 2011). Growth via formate oxidation and H_2_ production, representing one of the simplest forms of anaerobic respiration, has also been demonstrated in *Thermococcus* (Kim et al., 2010). The ability to use these low energy yielding metabolic pathways has been suggested to have important implications on survival in environments where energy supplies are at times transient or unavailable (Lee et al., 2008; Kim et al., 2010).

Biogeographic patterns have been observed in *Thermococcales* using Multilocus Sequence Typing (MLST) with populations of *Pyrococcus* from different regions shown to be genetically distinct (Escobar-Páramo et al., 2005). However, descriptions of population divergence and biogeography for *Thermococcus* have been inconclusive, therefore *Thermococcus* are described as being widespread and ubiquitous in hydrothermal habitats (Holden et al., 2001; Huber et al., 2006). Although there has been evidence suggestive of a correlation among *Thermococcus* diversity, environmental conditions, and geography (Holden et al., 2001; Summit and Baross, 2001; Huber et al., 2006; Teske et al., 2009), no strong biogeographic pattern has emerged. Analysis of *Thermococcus* by random amplified polymorphic DNA (RAPD) has identified diverse genomic profiles from the same sample site and similar profiles from different sites, illustrating both the biodiversity present and dispersal potential within a hydrothermal vent field (Lepage et al., 2004). While *Thermococcus* are commonly found in hydrothermal systems, the lack of evidence for biogeographic patterns has made it unclear whether populations are panmictic in their distribution or whether there is population structure at varying geographic scales.

With the exception of RAPD analysis, the genetic markers used in previous *Thermococcus* studies have not had a high degree of resolution. Two different and yet complementary DNA typing methods that utilize multiple sites from across the genome are MLST and Amplified Fragment Length Polymorphism (AFLP) analysis. MLST is a well-established DNA typing method using multiple loci spanning the genome to construct robust phylogenetic relationships among related microorganisms (Maiden et al., 1998). By applying MLST analysis to *Thermococcus* isolates their evolutionary relationships and biodiversity can be described more accurately, e.g., MLST analysis has delineated biogeographic patterns in *Sulfolobus* isolates sharing 99.8% sequence similarity for the SSU rRNA gene (Whitaker et al., 2003). MLST data have also provided a basis for defining species level boundaries through the comparison of loci and their average nucleotide identity or ANI (Konstantinidis and Tiedje, 2005). ANI comparisons of MLST loci, with sequence similarities of 95% or greater, have been correlated with species level ANI values determined from whole-genome comparisons in bacteria (Konstantinidis and Tiedje, 2005; Konstantinidis et al., 2006a,b).

In contrast to MLST analysis, AFLP analysis utilizes genome-wide restriction fragment lengths giving AFLP a higher discriminatory power allowing for the typing of microorganisms to the strain level (Lin et al., 1996; Olive and Bean, 1999; Savelkoul et al., 1999). AFLP genome fingerprints may vary as a result of nucleotide sequence divergence as well as the movement of transposable elements, insertions or deletions, or genome rearrangements. Due to these large scale genetic changes occurring at higher rates in comparison to nucleotide sequence divergence, AFLP fingerprints have the potential to resolve more recent genomic differentiation (Rademaker et al., 2000). Conserved loci used for MLST analysis are representative of the core genome while the genome-wide restriction fragments used in AFLP analysis may be more representative of variable regions associated with the dispensable or flexible genome, where gene acquisition and loss occurs more frequently (Medini et al., 2005; Tettelin et al., 2008; Cordero and Polz, 2014).

To address questions of biogeography, biodiversity and evolution in the oceanic crust, *Thermococcus* isolates from different hydrothermal regions were analyzed using MLST and AFLP. *Thermococcus* isolates from the Juan de Fuca Ridge (JdF) and East Pacific Rise (EPR) were the primary focus of this study with type strains and isolates from other hydrothermal habitats included for comparison.

## Materials and methods

### *Thermococcales* isolates and type strains

**Figure 1:**
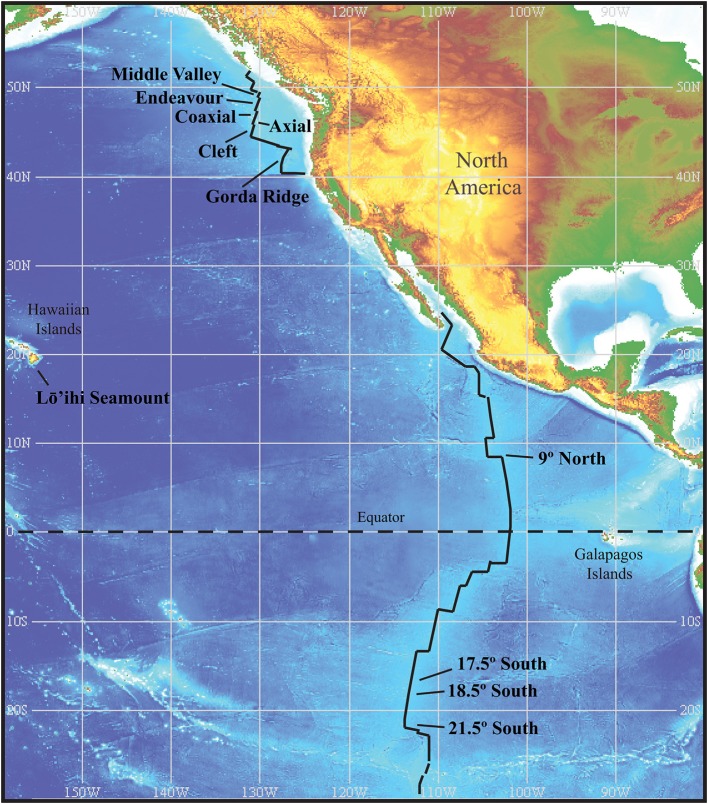
**Location of vents within the two main regions sampled**. The Juan de Fuca Ridge and associated vent segments with the Gorda Ridge to the south, and the East Pacific Rise. Sample sites within these two regions are at comparable distances from one another and provide nested sampling within regions. Image reproduced from the GEBCO world map 2014, www.gebco.net.

Sample material was collected from hydrothermal vent sites during research cruises between the years of 1988–2008. Study sites and sampling are as previously described (Holden et al., 2001; Summit and Baross, 2001; Huber et al., 2006; Davis and Moyer, 2008). Both submersible and remotely operated vehicles (ROV's) were used to collect a diversity of sample material that included plume samples, hot fluids, diffuse fluids, chimney walls, sulfide muds, and Alvinellid polychaete tissue samples. Sample material was used to inoculate liquid media for the enrichment of *Thermococcales*. Media formulations and methods are as previously described (Holden et al., 2001). Isolates in this culture collection were previously characterized through analysis of the SSU rRNA for genus level associations (Holden et al., 2001; Summit and Baross, 2001; Huber et al., 2006). The collection of 90 *Thermococcus* isolates contains representatives from the Juan de Fuca Ridge (JdF), Gorda Ridge, East Pacific Rise (EPR), Mid Atlantic Ridge (MAR), Mariana Arc and Loihi Seamount (Table 1). Sample sites within the JdF and EPR are at similar spatial distances providing nested sampling within these two regions (Figure 1). Distances among vents within regions range from ~65 to ~450 km, with distances between the main study sites, the JdF and EPR, up to ~7000 km. *Thermococcus* isolates and type strains from other regions, as well as *Pyrococcus* type strains, were included for comparison. Table S1 lists *Thermococcus* type strains included in AFLP and MLST analysis. Cultures of the type strains *Thermococcus kodakarensis* (JCM 12380) and *Thermococcus onnurines* (JCM 13517) were acquired through the Riken BioResource Center (Ibaraki, Japan) and were cultured in the same manner as other isolates in the collection. Genomic DNA for the type strains *Thermococcus barophilus* and *Thermococcus peptonophilus* were acquired from the American Type Culture Collection (Manassas, VA).

**Table 1:** *****Thermococcus*** isolates analyzed through AFLP and MLST and their corresponding vent segments (***n*** = 90)**.

**Juan de Fuca Ridge**	**55**	**East Pacific rise**	**22**	**Gorda Ridge**	**5**
Middle valley	8	9° North	1	**Lō'ihi Seamount**	**4**
Endeavor segment	23	17.5° South	7	**Mid Atlantic Ridge**	**2**
Coaxial segment	4	18.5° South	5	**Mariana Arc**	**2**
Axial volcano	18	21.5° South	9		
Cleft segment	2				

*Vent regions are in bold, vent segments are included as subsets for the two primary regions under study*.

### DNA extraction

Genomic DNA (gDNA) was extracted from freshly cultured cells. Cultured isolates were initially centrifuged at 750 × g for 5 min to pellet the sulfur. Supernatant was removed and centrifuged at 11,000 × g for 10 min in a chilled rotor (4°C). The resulting cell pellet was used for gDNA extraction with the DNeasy Tissue Kit (Qiagen, Valencia, CA) following manufacturer's protocols. A NanoDrop ND-1000 spectrophotometer (Thermo Scientific, Wilmington, DE) was used to check DNA concentration and purity.

### AFLP reactions and cluster analysis

AFLP analysis was performed using the Applied Biosystems (ABI) AFLP Microbial Fingerprinting kit (Applied Biosystems, Carlsbad, CA). Reactions and PCR conditions were as described in kit protocols using the restriction enzymes EcoRI and MseI. Primers were designed for the selective amplification of restriction fragments of between 50 and 500 base pairs. Two selective primer sets were used in separate reactions to obtain two individual AFLP profiles (EcoRI-0 and MseI-CT; EcoRI-C and MseI-G). AFLP reactions were purified across Sephadex G-75 columns and dried down in a 96 well plate before being resuspended in 15 μl of a 1:30 dilution of Liz-500 (ABI) size standard in formamide. Fragment lengths were analyzed using an ABI Prism 3130XL Genetic Analyzer. Electropherograms were optimized with samples rerun or diluted when necessary for comparable peak heights among isolates. Electropherogram data were checked for quality using ABI Genemapper software, and imported into BioNumerics version 4.61 (Applied Maths, Sint-Martens-Latem, Belgium) for further analysis.

Cluster analysis of AFLP electropherogram data was performed using the Pearson product-moment correlation coefficient in BioNumerics (Häne et al., 1993). Cluster analyses of AFLP primer sets were combined and averaged to construct a dendogram with cophenetic correlation coefficients.

### Multi locus sequence typing

Primers for MLST analysis were designed using nucleotide alignments from the following six annotated genomes: *Thermococcus kodakaraensis* KOD1 (Fukui et al., 2005), *Thermococcus onnurineus* NA1 (Lee et al., 2008), *Thermococcus gammatolerans* EJ3 (Zivanovic et al., 2009), *Thermococcus* sp. AM4 (Oger et al., 2011), *Thermococcus barophilus* MP (Vannier et al., 2011), and *Thermococcus sibiricus* (Mardanov et al., 2009). Gene sequences retrieved through GenBank were aligned with ClustalW multiple sequence aligner using default parameters (Thompson et al., 1994). Candidate genes were screened for conserved regions suitable for the amplification of between 300 and 500 base pairs. Loci associated with information processing and metabolism were the focus in identifying MLST candidate genes, along with the distribution of loci across the genome. Gene loci positions in the six reference genomes were plotted to check distribution and compare gene synteny (Figure S1). A portion of the following seven loci were picked for MLST analysis: SSU rRNA, Elongation Factor 1 Alpha subunit, DNA topoisomerase VI Alpha subunit, DNA polymerase II large subunit, threonyl-tRNA synthetase, pyruvate ferredoxin oxidoreductase Beta subunit, and histone acetyltransferase. The primer design software Primer3 was used to design primers (Rozen and Skaletsky, 2000). Degenerate primers were designed for all loci, with the exception of the SSU rRNA gene, in order to account for the diversity present in the six reference genomes (Table S2).

The following PCR mix was used for the SSU rRNA and EF1α genes: 50 ng of gDNA template, 0.5 μl JumpStart Taq (2.5 U/μl; Sigma, St. Louis, Mo), 1X Taq PCR buffer, 1.5 mM MgCl_2_, 0.2 mM each deoxynucleoside triphosphate, 0.2 μM each forward and reverse primer, and molecular grade water to a total volume of 50 μl for each reaction. For the SSU rRNA genes the following PCR cycle was used: an initial 10 min hot start at 95°C, followed by 30 cycles of denaturation (95°C for 30 s), annealing (58°C for 30 s), and elongation (72°C for 30 s). This was followed by a final elongation step at 72°C for 7 min. For the EF1α genes the PCR cycle was the same as the above with the exception of a 56°C annealing temperature. For the remaining five genes the mixture and conditions were the same as the above with the exception of a 0.8 μM concentration for forward and reverse primers, and 55°C annealing temperature.

PCR amplicons were verified and checked for size through gel electrophoresis. Amplicons were sequenced using ABI BigDye Terminator v3.1, using an ABI Prism 3130XL Genetic Analyzer. Nucleotide sequences were contiguously assembled using BioNumerics and were verified via BLASTn (Altschul et al., 1990). The concatenated alignment of all seven amplicons following the masking of sequence data and removal of non-homologous positions resulted in 2648 bp of nucleotide sequence for analysis. Partial sequences for all loci were submitted to GenBank and assigned accession numbers (SSU rRNA: KP187908-KP187997, DNA polymerase II large subunit: KP187998-KP188087, DNA topoisomerase VI subunit A: KP188088-KP188177, elongation factor-1 alpha: KP188178-KP188267, histone acetyltransferase: KP188268-KP188357, pyruvate:ferredoxin oxidoreductase subunit beta: KP188358-KP188447, threonyl-tRNA synthetase: KP188448-KP188537).

The ExPASy translate tool (Swiss Institute of Bioinformatics) was used to determine the correct reading frame for protein coding genes before nucleotide sequences were translated into amino acid sequences using MEGA v5 (Tamura et al., 2011). Sequences of individual loci were aligned by ClustalW in MEGA v5 with all gaps removed.

A sequence based test for selection was performed on each protein coding locus using the Datamonkey online server using the following parameters: Data type was codon, genetic code set to universal code, and the method used was SLAC (Pond and Frost, 2005). Non-synonymous verses synonymous (dN/dS) ratios were calculated on the in-frame nucleotide alignments of individual loci to determine if loci were under strong selection.

### Phylogenetic analysis

Maximum likelihood (ML) phylogenetic trees were constructed using the RAxML BlackBox online server (Stamatakis et al., 2008). A mixed/partition model was applied to the concatenated alignment of nucleotide (SSU rRNA) using the CAT model (Stamatakis, 2006; Stamatakis et al., 2008) and amino acid sequences, using the WAG (Whelan and Goldman, 2001) protein model, with per gene optimization of branch lengths and 100 bootstraps. The tree with the lowest log likelihood score was selected from 10 replicates. The concatenated ML tree was rooted using the *Crenarchea, Staphylothermus marinus* as an outgroup. Homologs in *Staphylothermus marinus* for six of the seven loci (no suitable homolog was found for DNA polymerase II) were used for phylogenetic analysis and rooting of the tree using the parameters described above. Clades were assigned to groupings of three or more isolates. Bootstrap values of 20 and above were reported. The ML tree for the SSU rRNA locus, with the inclusion of *Palaeococcus ferriphilus* and *Palaeococcus helgesonii* sequences (Takai et al., 2000; Amend et al., 2003), was also rooted using *Staphylothermus marinus* as an outgroup. For both trees, the choice of *Staphylothermus marinus* as an outgroup was made as it required minimal masking relative to the several other *Crenarcheaota* tested without changing position of the root.

### MLST clade analysis

The 2648 bp concatenated nucleotide sequence was used for analysis of the Average Nucleotide Identity (ANI) of clades, as an estimate of species level boundaries (Konstantinidis and Tiedje, 2005; Konstantinidis et al., 2006a,b). ANI values were calculated through BLAST (bl2seq; NCBI BLAST) with the lowest similarity value in comparisons between isolates recorded as the ANI for a particular clade.

The six protein-coding loci were used for the analysis of GC-content of clades, an estimate of evolutionary history, using the Datamonkey online server as described above (Pond and Frost, 2005).

Linkage analysis was performed to test for evidence of recombination in MLST loci. In clonal organisms linkage among loci (linkage disequilibria) is expected with evidence of unlinked loci (linkage equilibria) associated with gene transfer or recombination events. Linkage among loci was tested using the Non-redundant database (NRDB; PubMLST) and Linkage Analysis (LIAN) v3.5 set to default parameters, e.g., Monte Carlo with 100 iterations. (Haubold and Hudson, 2000). Isolate loci were coded through NRDB and the null hypothesis of linkage equilibrium for clades was tested using LIAN v3.5 with the standardized index of association (*I*_A_) reported.

### Codon GC-content

Ratios of the GC-content for first and third codon positions were analyzed as a measure of gene history (Muto and Osawa, 1987). A two dimensional plot of codon ratios was constructed from the average GC-content of all six protein coding loci. The first and third codon positions of loci were calculated for individual clades and individual isolates and type strains when not associated with Clades I-X using MEGA v5 (Tamura et al., 2011). The first and third codon positions were plotted in a similar manner previously shown to differentiate genomic variation in bacteria (Kaplan and Fine, 1998).

### Mantel test

A Mantel test comparing geographic distance and genetic distance was performed using the statistical software zt (Bonnet and de Peer, 2002). Pairwise genetic distances for nucleotide sequence data were calculated using the Maximum Composite Likelihood model in MEGA v5 (Tamura et al., 2011). Geographic distances among vents were calculated from hydrothermal vent latitude and longitude. Matrices of isolate genetic and geographic distance were compared to test the null hypothesis of independence between matrices. A simple Mantel test with 10,000 randomizations was performed on all isolates, on isolates from the two main regions being investigated (JdF and EPR) and on closely associated phylogenetic clades.

### Analysis of molecular variance

Analysis of Molecular Variance (AMOVA) was calculated in Arlequin version 3.11 (Excoffier et al., 2005) on the concatenated nucleotide sequences of all seven loci. AMOVA was used to test for correlations between sample type and sample site. Isolates were grouped by the sample type they were isolated from and by the hydrothermal vent site from which they were isolated, with the *p*-value significance test for variance carried out using 10,000 permutations.

### Principal component analysis

Principal Component Analysis (PCA) was performed on AFLP band calling data using BioNumerics v4.61. Band calling data were collected through the automated selection of bands from both primer sets using the following parameters: a minimum profiling of 5% for primer set 1, a minimum profiling of 10% for primer set 2, with the optimization and position tolerances for selecting bands set to 0.10% for both primer sets. Band calling resulted in an average of 14 bands per isolate for primer set 1 and an average of 15 bands per isolate for primer set 2. Band calling data from both primer sets were combined and converted into a binary presence absence matrix. Default settings were applied for PCA in BioNumerics, subtracting the average for characters.

## Results

### AFLP cluster analysis

Through cluster analysis, isolate AFLP profiles are grouped by similarity. The cluster analysis dendogram topology is well supported by cophenetic correlation coefficients (Figure S2). Isolates from the JdF and EPR are differentiated at regional levels with no clustering of isolates between these two regions. Isolate diversity is exemplified by the number of clusters found within the JdF and EPR regions with up to five clusters identified for each. These clusters are made up of isolates from more than one vent site within a region, more than one sample type and for JdF isolates, from different sampling years. Isolate groupings illustrate the dispersal potential between vent sites within a region with clusters containing isolates from vents spread throughout a region. Isolates previously identified through SSU rRNA analysis as *Pyrococcus* clustered together with the exception of isolate MV7. Type strains and isolates from regions other than the JdF or EPR had low AFLP profile similarity with isolates in this study, with the exception of the type strain *Thermococcus onnurines*. This type strain (*T. onnurines*) was isolated from the Papua New Guinea-Australia-Canada-Manus (PACMANUS) field (Bae et al., 2006) and had high similarity with the cluster containing Gorda Ridge isolates and isolate CX3 from the coaxial segment, with ≥ 68% AFLP profile similarity among these isolates.

### MLST and phylogenetic analysis

Analysis of the individual alignments for the six protein coding loci did not show significant evidence for any of the loci undergoing strong selection (Table S3), making these loci suitable for MLST analysis. Ratios of dN/dS were consistent with dN/dS ratios reported for conserved genes under purifying selection (Kuhn et al., 2006).

**Figure 2:**
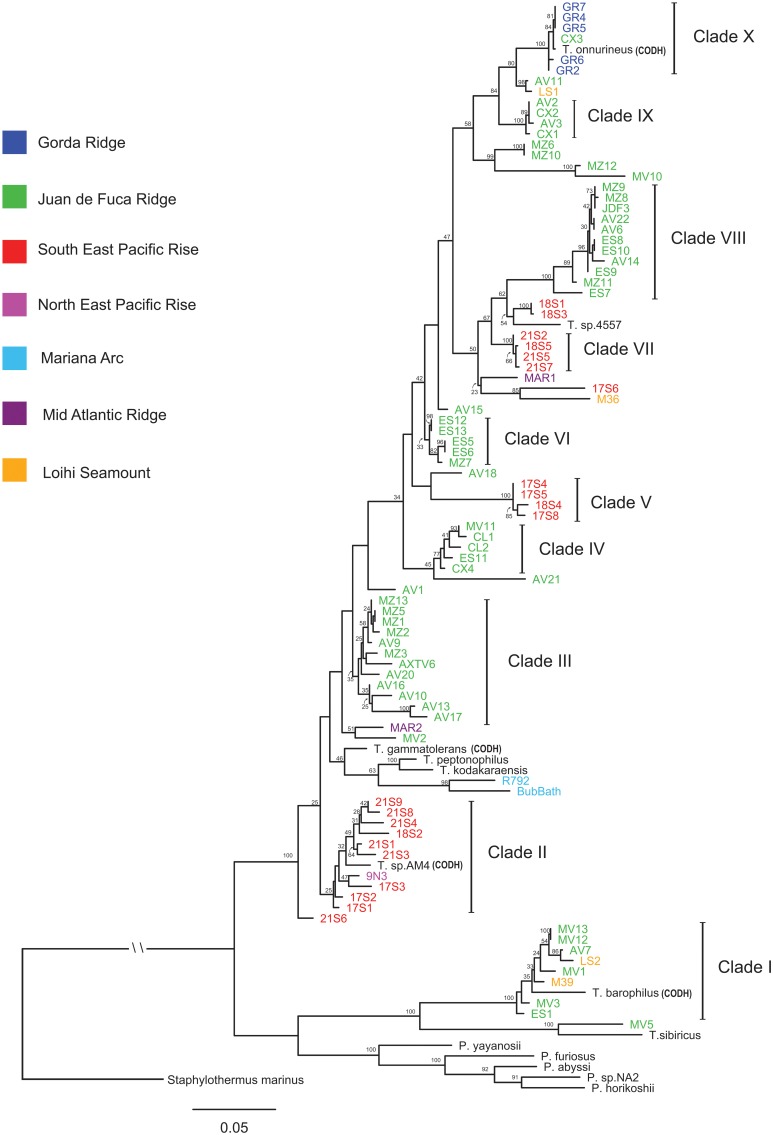
**Maximum likelihood phylogenetic tree of the concatenated amino acid and SSU rRNA sequences constructed using RAxML with a mixed/partitioned model and per gene optimization of branch lengths with 100 bootstraps**. Bootstrap values of 20 and above are reported. The *Crenarchaeota Staphylothermus marinus* was used as an outgroup. Genomes containing carbon monoxide dehydrogenase genes are labeled CODH.

The ML phylogeny of concatenated MLST loci differentiated isolates into clades that are in general agreement with isolate groupings through AFLP cluster analysis (Figure 2 and Figure S2). Isolates from the JdF and EPR are differentiated into lineages that are phylogenetically related across these two regions (Figure 2). The regional divergence among phylogenetically related isolates is most apparent in the paraphyly observed between Clades VII and VIII. A general pattern of isolates differentiated by region was observed with individual clades made up of isolates from that same region. Regional groupings were most apparent in the isolates associated with the JDF and EPR; however, regional groupings were also observed in the two isolates from the Mariana Arc, the type strains *T. peptonophilus* and *T. kodakaraenis* both from Japan, and the type strains *T. sp*. 4557 and *T. sp*. AM4 isolated from the EPR (which both group with lineages also from the EPR). Exceptions to regional groupings are seen in Clades I and X, a pattern also observed through AFLP cluster analysis. Clade I contains isolates from the JdF Ridge, Loihi Seamount, and the type strain *T. barophilus* from the Mid Atlantic Ridge (MAR). Clade X contains isolates from the Gorda Ridge, an isolate from the CoAxial Segment (of the JdF), and the type strain *T. onnurines* from the PACMANUS Basin, with all of these isolates sharing 99% sequence identity. *Pyrococcus* type strains along with Clade I, the type strain *T. sibiricus* and isolate MV5 are placed in a basal position in the phylogenetic tree, ancestral to Clades II through X. The type strain *P. yayanosii* has the shortest distance, from these basal groups, to *Thermococcus* isolates in Clades II through X. The ancestral relationship between *Pyrococcus* type strains and *Thermococcus* isolates in the ML phylogeny of concatenated MLST loci remains unresolved.

**Figure 3:**
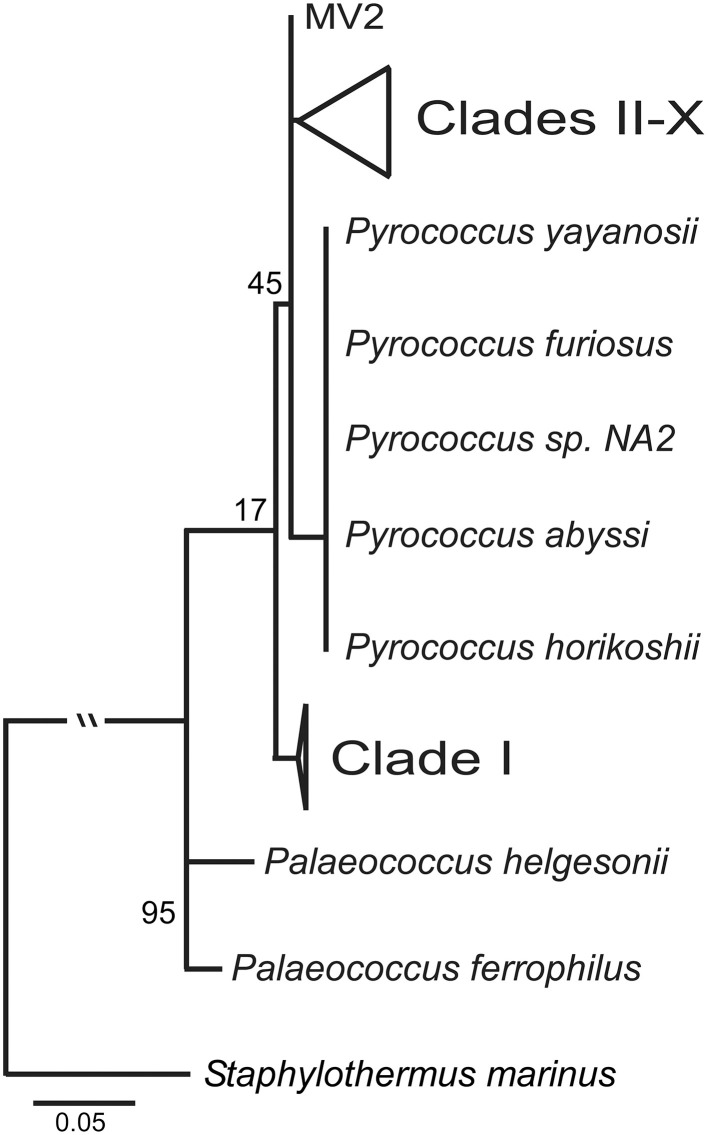
**Maximum likelihood phylogenetic tree of the SSU rRNA with the inclusion of ***Palaeococcus*** species and with ***Staphylothermus marinus*** as an outgroup**. Clade I is ancestral to both *Pyrococcus* type strains and *Thermococcus* isolates in clades II–X. Numbers at nodes represent bootstrap values.

The ML tree for the SSU rRNA gene, which includes *Palaeococcus* isloates and is rooted with *Staphylothermus marinus*, places the Clade I *Thermococcus* isolates in an ancestral position to *Pyrococcus* type strains and the *Thermococcus* isolates found in Clades II through X (Figure 3). This illustrates the paraphyletic associations among members of the *Thermococcus* and *Pyrococcus* genera. Several other *Crenarchaeota* were tested as an outgroup including: *Cenarchaeum symbiosum, Sulfolobus tokodaii, Nitrosopumilus maritimus*, and *Pyrobaculum aerophilum*. None of these other taxa changed the position of the root (data not shown).

### MLST clade analysis

Analysis of clade ANI allowed for clade diversity to be estimated based on species level boundaries observed in bacteria (ANI ≥ 95%). Clades I, II, and III have ANI values below 95% reflecting diversity that is likely beyond the individual species level (Table 2). Clades IV through X have ANI values at or above 95% suggesting that these clades may represent individual species. Pairwise comparisons among all clades (data not shown) result in ANI values < 95%, suggesting that individual clades represent distinct species.

**Table 2:** **Clade analysis for Average Nucleotide Identity (ANI), GC-content and test for linkage among loci with the Index of association (***I***_***A***_) and probability value reported**.

**Clade**	**ANI%**	**GC ratio**	***I*_A_**	***p*-value**
I	91	45.50	0.3667	0.001
II	94	57.65	0.0189	0.394
III	93	57.97	0.4845	0.001
IV	98	59.05	−0.0352	0.605
V	97	57.43	0.6179	0.001
VI	99	58.49	0.5873	0.001
VII	99	59.03	0.3644	0.001
VIII	95	59.64	0.0328	0.248
IX	99	57.03	0.1061	0.251
X	99	55.93	0.6356	0.001

Variation in GC-content was observed among all clades (Table 2). Clade I at 45.50% had the lowest GC-content in comparison to other *Thermococcus* clades and was closer to GC values reported for *Pyrococcus* genera (Lecompte et al., 2001). Clade X with a GC-content of 55.93% had the next lowest percentage with clades II through IX having GC-content ratios between 57.03 and 59.64%.

Linkage analysis was applied to test for linkage among the MLST loci. Linkage analysis of individual clades rejected the null hypothesis of linkage equilibria, with the measure of linkage (*I*_A_) significantly different from zero and/or not significant. Therefore, clade analysis did not show evidence of gene transfer or recombination in MLST loci examined.

### Codon GC-content ratios

A two dimensional plot of average GC-content at the first and third codon position illustrates differences in codon GC ratios for clades, isolates and type strains from the phylogenetic tree (Figure S3). *Thermococcus* isolates from Clade I along with the type strain *Thermococcus sibiricus* and isolate MV5 have codon GC ratios that are closer to *Pyrococcus* isolates and type strains, in agreement with our MLST phylogenetic tree (Figure 2). *Pyrococcus yayanosii*, which has been shown to have a higher GC content in comparison to other *Pyrococcus* (Jun et al., 2011), has a codon GC ratio closer to those calculated for the *Thermococcus* isolates found in Clades II through X.

### Mantel test

Mantel's analysis testing the correlation between genetic distance and geographic distance did not find a significant correlation when looking at all isolates or isolates from either the JdF or EPR regions separately (Table S4). The lack of a correlation at these scales is in part due to the degree of genetic diversity present within regions and within vent sites. Analysis of closely associated phylogenetic clades from the two main regions (JdF and EPR) did find a significant correlation between genetic distance and geographic distance. An exception to this is the analysis of Clades IX and X, where geographic distance and genetic distance are not correlated. In particular, Clade X contains isolates from disparate regions which share high sequence similarity.

### Analysis of molecular variance

Variation in isolates grouped by sample type (1.1% of variation *P* = 0.324) and by sample site (16.2% of variation *P* = 0.121) showed no significant correlation using an AMOVA of MLST nucleotide data.

### Principal component analysis

**Figure 4:**
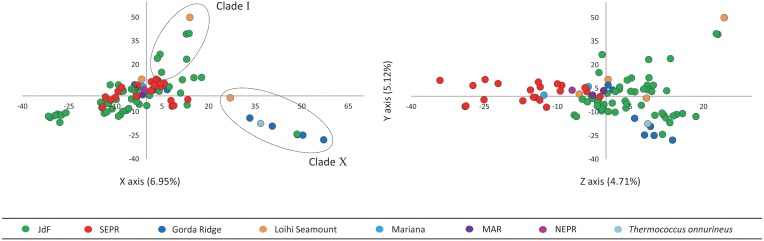
**Principal component analysis of AFLP data with the first three principal components describing the greatest variation plotted on the X, Y, and Z axes**. Principal component 1 and 2 plotted on the X and Y axes differentiate isolates in Clade I and Clade X from other isolates in this study. Principal component 3 plotted on the Z axis differentiates isolates into larger regionally related groups.

PCA revealed that the first three principal components described 16.8% of the variation in the AFLP data (Figure 4). Principal components 1 and 2 plotted on the X and Y axes respectively, differentiate isolates from Clade I and Clade X from other isolates in this study. Principal component 3 plotted on the Z axis differentiates isolates into larger regionally related groups, particularly the JdF isolates (northern-most) from the EPR isolates (southern-most).

## Discussion

A comparison of *Thermococcus* isolates through AFLP and MLST analysis have made biogeographic patterns evident; however, phylogenetic analysis does not suggest a simple model of allopatric speciation. This suggests that there is high dispersal in the *Thermococcus* genus. The strongest biogeographic patterns were observed between northern isolates from the JdF and southern isolates from the EPR, most likely due to these regions having the highest representation (Table 1).

A barrier to dispersal between northern and southern hydrothermal regions developed as the North American Plate overrode the Pacific Plate. The discontinuous nature of the plate boundary and associated hydrothermal vents allowed for endemic populations to develop over time between the northern JdF and southern EPR. The paraphyletic association between Clades VII and VIII demonstrates the shared ancestry between these two lineages and their divergence when populations became isolated from one another. The limited mixing of water masses through circulating ocean currents may have also contributed to the isolation of these populations (Müller et al., 2014). Analysis of *Thermococcus* isolates from a more contiguous series of hydrothermal venting regions, like the Mid Atlantic Ridge, would be useful in further resolving the relationship between dispersal and genetic distance in this genus.

The high dispersal observed in *Thermococcus* may result from an ability to survive outside of the hydrothermal vent environment for extended periods of time. Thermophilic activity has been measured in equatorial deep-sea sediments hundreds of km away from any known hydrothermal activity, thereby hypothesizing long distance transport and survival from the EPR (Dobbs and Selph, 1997). Dispersal over great distances must be considered particularly when looking at the isolates found in Clade X, where high sequence and AFLP profile similarity was found in isolates from vent sites ~9500 km apart (i.e., Gorda Ridge and Manus Basin). Explanations for the high similarity observed in these isolates may include: a population bottleneck or recent population divergence, increased dispersal potential resulting from the ability to survive outside of the vent environment, or possibly a niche-specific ecotype due to unique metabolic characteristics. Isolates in Clade X that were collected from the Gorda Ridge have already been shown to grow over an atypically broad range of temperatures (45–90°C) for hyperthermophiles (Summit and Baross, 1998). The discovery of the CODH gene cassette in the type strain *T. onnurines* (found in Clade X) and the occurrence of these genes in other distantly related type strains may be indicative of a gene transfer event providing a selective advantage. Isolates found in Clade X may also be representative of a subsurface ecotype, with five of the seven isolates in this clade isolated from a plume event (Summit and Baross, 1998). Hydrothermal fluids released from subseafloor reservoirs during a plume event are believed to have residence times of from months to years and hydrothermal fluids have been detected thousands of kilometers from their origin (Lupton, 1996; Lupton et al., 1998, 1999). This makes hydrothermal plumes a potential vehicle for the dispersal of microorganisms associated with subsurface hydrothermal habitats over great distances. Long distance dispersal has also been demonstrated in dormant thermophilic endospores through the circulation of ocean currents (Müller et al., 2014). Further investigation of Clade X will require full-genome comparisons against other *Thermococcus* genomes, to look for genes unique to this group that may allow for greater survival and dispersal outside the hydrothermal environment.

Clade I represents a group of *Thermococcus* that was unique in comparison to the other clades identified; however, Clade I like Clade X contained isolates from different regions (i.e., Loihi Seamount and Juan de Fuca Ridge). The most striking characteristic of the Clade I group was the phylogenetic distance between this group from other *Thermococcus* isolates as compared to their close proximity to several *Pyrococcus* type strains. This pattern was also observed in the GC-content analyses illustrating the divergence of the Clade I lineage with the *Pyrococcus* genus from the other *Thermococcus* isolates examined. In the SSU rRNA gene tree (Figure 3), Clade I was placed in an ancestral position relative to both *Pyrococcus* type strains and *Thermococcus* isolates from Clades II through X resulting in paraphyletic groupings of the *Thermococcus* and *Pyrococcus* genera. This topology has also been demonstrated in other phylogenetic analyses of the *Thermococcales* (Teske et al., 2009; Yilmaz et al., 2014). These data suggest that Clade I is a close sister lineage to *Pyrococcus* requiring reclassification.

Biodiversity in the *Thermococcus* genus has been made evident in this study through the identification of three or more lineages co-occurring within the same hydrothermal vent system. These divergent lineages have likely adapted to different ecological niches, allowing them to coexist at the same vent site. Previous research has shown a correlation between environmental habitat and phylogeny with the hydrothermal vent habitats of sulfide chimneys and subseafloor zones proposed as the differing environments involved in the maintenance of diversity (Summit and Baross, 2001). Although no significant correlation was found in our study between lineages and the environmental samples from which they were isolated, the broader divergence observed in the phylogenetic tree may well be due to the differing source habitats as previously described. These broader lineage groups, Clades II and III vs. Clades VII and VIII, may represent lineages adapted to different niches and present in both regions, which have diverged over time into regionally endemic populations. Determining the source environment for any of the lineages identified will be extremely difficult particularly when considering the continuous mixing of fluids within subsurface hydrothermal conduits and the limitations imposed by current sampling techniques. While it may not be possible to improve greatly on sampling techniques in the near future, the biodiversity described can be investigated more thoroughly through the genomic analysis of metabolic characteristics associated with different lineages and how these relate to the utilization of different habitats.

Horizontal gene transfer (HGT) and recombination are believed to play an important role in the mixing of microbial populations and shaping of diversity (Gogarten et al., 2002; Nesbø et al., 2006; Zhaxybayeva et al., 2006), however the role of HGT is unclear since there is a decrease in these events as geographic distance and genetic distance among microorganisms increase, e.g., extrinsic and intrinsic barriers to gene transfer and recombination (Whitaker et al., 2005; Reno et al., 2009; Cadillo-Quiroz et al., 2012). Genomic diversity in *Thermococcus* isolates was investigated through the analysis of AFLP and MLST data for evidence of genomic rearrangements and gene transfer events. The conserved nature of MLST loci evident in dN/dS ratios and through linkage analysis support the theory that conserved housekeeping genes making up the core genome are unlikely to undergo gene transfer or recombination (Medini et al., 2005; Tettelin et al., 2008; Cordero and Polz, 2014). The same lineages identified through MLST were also identified through AFLP, although AFLP analysis detected greater genomic variation. Analysis of AFLP data through PCA (Figure 4) provides evidence suggestive of regionally related recombination, with phylogenetically distinct lineages clustered into larger regionally related groups. This is in contrast to what is shown in the ML phylogenetic tree constructed from MLST data (Figure 2) where isolates from a shared region are often differentiated into distinct clades. Shared genomic characteristics within regions, between divergent lineages, may be detected in AFLP data as the genome wide restriction fragment lengths are more representative of variable regions of the genome where gene acquisition and loss occur more frequently.

Biogeographic patterns in viruses and mobile genetic elements have been identified in both terrestrial and marine environments as exemplified by *Sulfolobus* and *Vibrio*, respectively (Held and Whitaker, 2009; Boucher et al., 2011). In the *Thermococcales*, geographically associated recombination has been reported with the identification of a shared 16 kb region flanked by insertion sequence (IS) elements, found in *Pyrococcus furiosus* and *Thermococcus litoralis* both of which were isolated from Vulcano Island, Italy (Diruggiero et al., 2000). To address regionally related recombination in *Thermococcus*, mobile genetic elements like insertion sequence elements and transposons (Diruggiero et al., 2000; Zivanovic et al., 2002; Escobar-Páramo et al., 2005; Fukui et al., 2005; Mardanov et al., 2009) along with viral related elements (Fukui et al., 2005; Mardanov et al., 2009; Portillo and Gonzalez, 2009; Zivanovic et al., 2009; Vannier et al., 2011) and extracellular vesicles (Choi et al., 2015) can be investigated within populations at regional levels and across regions, for further evidence in support of regionally related recombination.

This study begins to elucidate the level of genomic resolution required to track population divergence in isolates of *Thermococcus* at marine hydrothermal systems. These systems are thermodynamically energized by strong gradients (e.g., temperature, oxygen, etc.) fueling multiple potential habitats within the subsurface crustal environment (Edwards et al., 2011; Orcutt et al., 2011). The ability to observe divergence in microorganisms is largely dependent on the geographic scale investigated and level of genetic resolution applied. Biogeographic patterns shown in the archaeal genus *Thermococcus* demonstrate that even in microorganisms with dispersal over thousands of kilometers, divergence can occur when populations are isolated from one another. By identifying the appropriate scale and level of resolution required, the impact that geography and barriers to dispersal have on shaping microbial diversity are now being realized. Identifying the role and significance of mobile genetic elements in the shaping of microbial populations is the next step to understanding the evolutionary forces behind the planet's vast microbial diversity.

### Conflict of interest statement

The authors declare that the research was conducted in the absence of any commercial or financial relationships that could be construed as a potential conflict of interest.
