# Supplementary material for: Biogeography and evolution of Thermococcus isolates from hydrothermal vent systems of the Pacific
Source: Front Microbiol. 2015 Sep 24;6:968. doi: 10.3389/fmicb.2015.00968 (PMC4585236; doi:10.3389/fmicb.2015.00968)
Supplement: Supplementary file 1 [file Table1.PDF]

Table S1. *Thermococcus* type strains analyzed by AFLP and MLST.

**Type Strains (n = 8)**		AFLP	MLST
Thermococcus kodakerensis	Kagoshima, Japan	x	x
Thermococcus peptonophilus	Izu - Bonin forearc	x	x
Thermococcus onnurines	PACMANUS field of the East Manus Basin	x	x
Thermococcus barophilus	Mid-Atlantic Ridge	x	x
Thermococcus gammatolerans	Guaymas basin		*
Thermococcus sibiricus	Oil reservoir in Western Siberia		*
Thermococcus sp. AM4	East Pacific Rise 13 deg. North		*
Thermococcus sp. 4557	Guaymas basin		*

* sequence data retrieved from GenBank
