# Supplementary material for: Biogeography and evolution of Thermococcus isolates from hydrothermal vent systems of the Pacific
Source: Front Microbiol. 2015 Sep 24;6:968. doi: 10.3389/fmicb.2015.00968 (PMC4585236; doi:10.3389/fmicb.2015.00968)
Supplement: Supplementary file 2 [file Table2.PDF]

Table S2. MLST primer sequences.

Sequence Name	Bases	Sequence	GC Content	Tm
DNA polymerase II large subunit Forward	20	GAR GAR TGG TGG RTT CAG GA	52.50	55.31
DNA polymerase II large subunit Reverse	20	ATC CAR CTT ATH CCC CKR TC	49.17	53.39
Pyruvate ferredoxin oxidoreductase beta subunit Forward	20	GAR GAC AAG CCV AAG AAG TG	50.83	54.02
Pyruvate ferredoxin oxidoreductase beta subunit Reverse	22	CTT GAA DAG GTG CTT RAA YCT K	40.15	52.17
Threonyl tRNA synthetase Forward	20	GCC MGA TAT GCA YAC SGT HG	56.67	56.74
Threonyl tRNA synthetase Reverse	20	TGW ATB GGR CTG AGC CAK AG	53.33	55.83
DNA topoisomerase VI alpha subunit Forward	21	AAG CMT AYT AYG CSA ACA AGC	45.24	54.63
DNA topoisomerase VI alpha subunit Reverse	20	ATR TCR TCC ATN GTC ATK CC	45.00	52.23
Histone acetyltransferase Forward	21	AGT GGG TTM GSG THA TGA GRA	49.21	56.48
Histone acetyltransferase Reverse	19	CVC KRT GCT GCC ACT CRT A	58.77	57.88
Elongation factor 1 alpha subunit Forward	23	MGT YAA GAA GAG CGA CAA GAT GC	47.80	56.70
Elongation factor 1 alpha subunit Reverse	21	CAC CGG TCT TGA TGA AYT GYG	52.30	56.30
SSU rRNA Forward	20	GGG GTC CGA CTA AGC CAT GC	65.00	60.60
SSU rRNA Reverse	21	GAT TTC GCC AGG GAC TTA CGG	57.10	58.00
