# Supplementary material for: Biogeography and evolution of Thermococcus isolates from hydrothermal vent systems of the Pacific
Source: Front Microbiol. 2015 Sep 24;6:968. doi: 10.3389/fmicb.2015.00968 (PMC4585236; doi:10.3389/fmicb.2015.00968)
Supplement: Supplementary file 3 [file Table3.PDF]

Table S3. dN/dS ratios calculated for MLST protein coding loci as a measure of selection. Loci dN/dS values reflect the conservation of these genes under purifying selection.

Gene Locus	dN/dS
DNA Polymerase II	0.1106
DNA Topoisomerase VI	0.0199
Elongation Factor 1α	0.0623
Histone Acetyltransferase	0.0483
Pyruvate Ferredoxin Reductase	0.0464
Threonyl tRNA Synthetase	0.0371
