# Supplementary material for: Biogeography and evolution of Thermococcus isolates from hydrothermal vent systems of the Pacific
Source: Front Microbiol. 2015 Sep 24;6:968. doi: 10.3389/fmicb.2015.00968 (PMC4585236; doi:10.3389/fmicb.2015.00968)
Supplement: Supplementary file 4 [file Table4.PDF]

Table S4. Mantel Test for selected isolates. Phylogenetically related clades from different regions show a significant correlation between genetic distance and geographic distance with the exception of Clades IX and X.

**Mantel Test**	**r^2**	**p-value**
All Isolates	0.016	0.0229
Juan De Fuca Isolates	0.007	0.0425
South East Pacific Rise Isolates	0.006	0.1186
Clade I	0.312	0.0511
Clades II & III	0.719	0.0001
Clades IV, V & VI	0.603	0.0003
Clades VII & VIII	0.699	0.0001
Clades IX & X	0.008	0.3579
