# Supplementary material for: Biogeography and evolution of Thermococcus isolates from hydrothermal vent systems of the Pacific
Source: Front Microbiol. 2015 Sep 24;6:968. doi: 10.3389/fmicb.2015.00968 (PMC4585236; doi:10.3389/fmicb.2015.00968)
Supplement: Supplementary file 5 [file Image1.PDF]

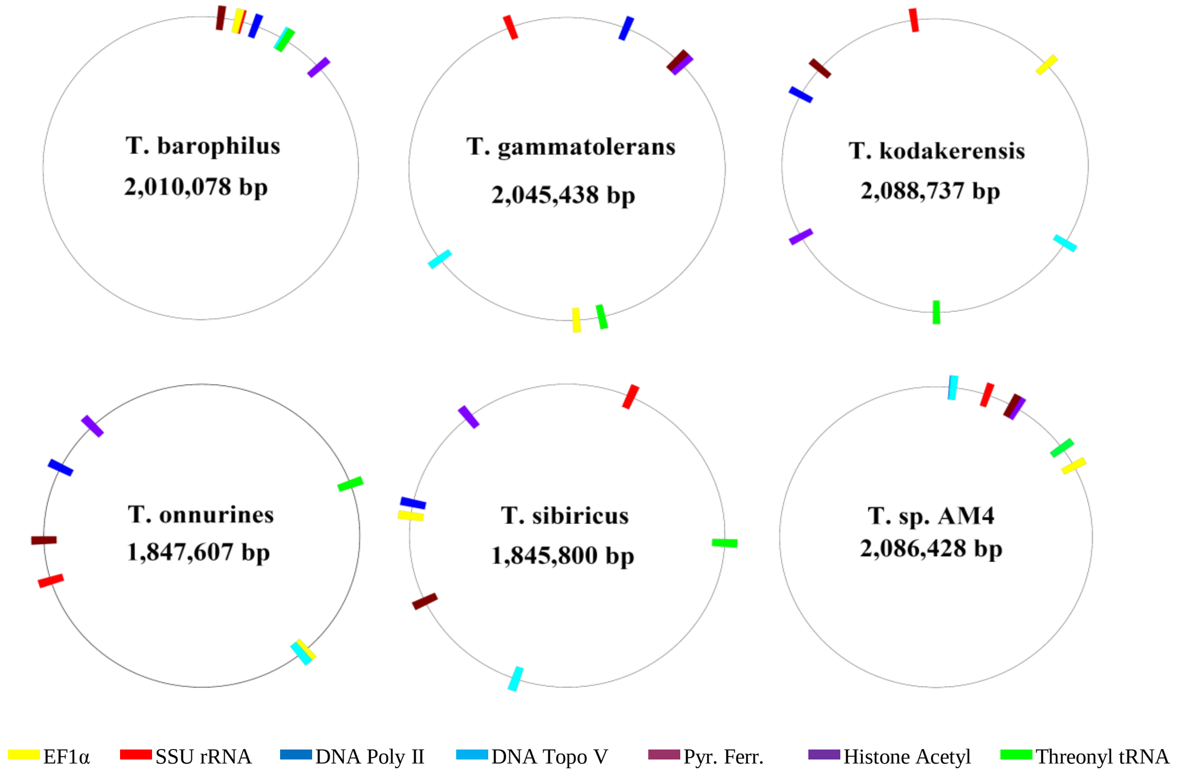

Figure S1. Gene loci maps for the six *Thermococcus* reference genomes used for MLST primer design. Loci maps illustrate the genomic rearrangements and reduced gene synteny among *Thermococcus* species.
