# Supplementary material for: Biogeography and evolution of Thermococcus isolates from hydrothermal vent systems of the Pacific
Source: Front Microbiol. 2015 Sep 24;6:968. doi: 10.3389/fmicb.2015.00968 (PMC4585236; doi:10.3389/fmicb.2015.00968)
Supplement: Supplementary file 6 [file Image2.PDF]

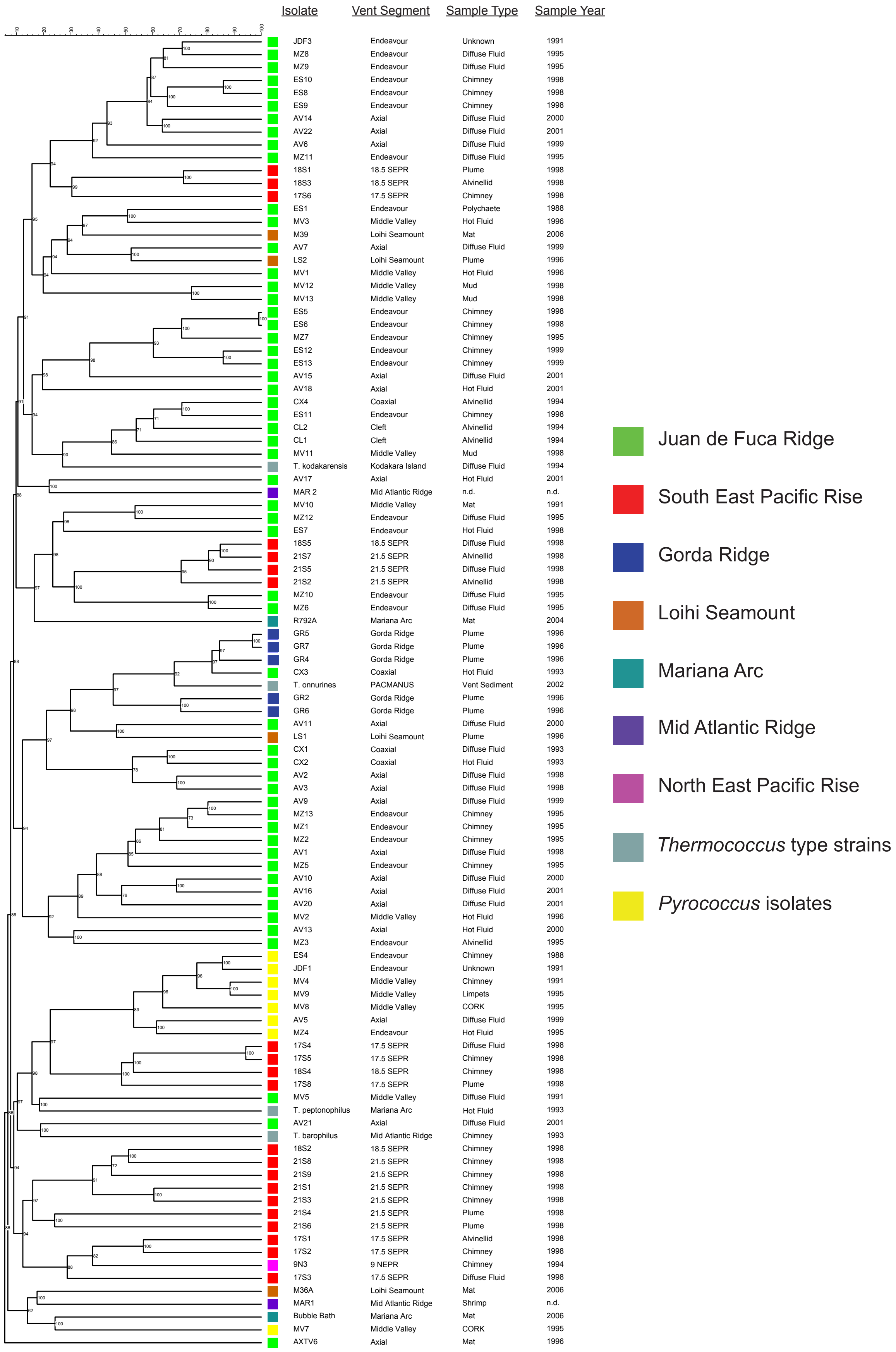

Figure S2. Cluster analysis of AFLP data through the Pearson product-moment correlation coefficient and UPGMA methods. Isolates are clustered into regionally related groups consisting of isolates from varying sample types and sample sites. Scale bar is Pearson product moment correlation r-value X 100. Numbers at nodes are cophenetic correlation coefficients.
