# Supplementary material for: Biogeography and evolution of Thermococcus isolates from hydrothermal vent systems of the Pacific
Source: Front Microbiol. 2015 Sep 24;6:968. doi: 10.3389/fmicb.2015.00968 (PMC4585236; doi:10.3389/fmicb.2015.00968)
Supplement: Supplementary file 7 [file Image3.PDF]

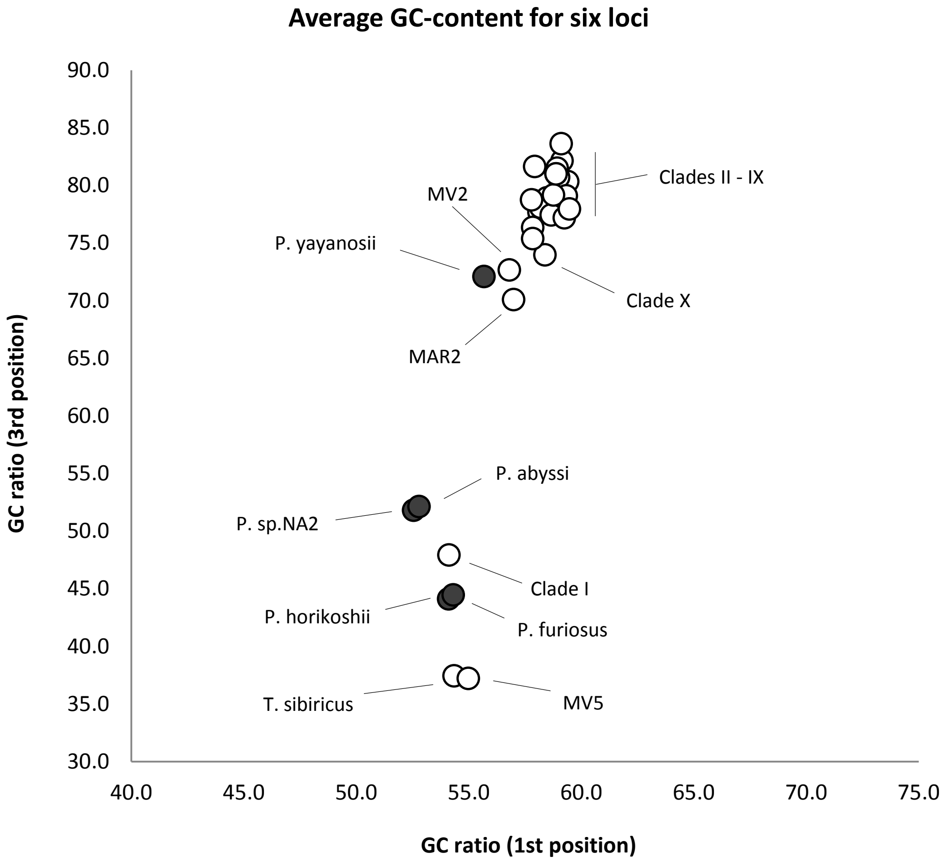

Figure S3. Average G+C ratio for first and third codon positions of six protein coding loci. Averages were taken for clades and for individual isolates or type strains when not associated with Clades I through X. Closed circles are *Pyrococcus* and open circles are *Thermococcus*.
